# Breath Analysis for Early Detection of Malignant Pleural Mesothelioma: Volatile Organic Compounds (VOCs) Determination and Possible Biochemical Pathways

**DOI:** 10.3390/cancers12051262

**Published:** 2020-05-16

**Authors:** Alessia Di Gilio, Annamaria Catino, Angela Lombardi, Jolanda Palmisani, Laura Facchini, Teresa Mongelli, Niccolò Varesano, Roberto Bellotti, Domenico Galetta, Gianluigi de Gennaro, Sabina Tangaro

**Affiliations:** 1 Department of Biology, University of Bari Aldo Moro, 70126 Bari, Italy; facchini.laura@gmail.com (L.F.); teresamongelli@libero.it (T.M.); gianluigi.degennaro@uniba.it (G.d.G.); 2 Apulian Breath Analysis Center (CeRBA), IRCCS Giovanni Paolo II, 70124 Bari, Italy; a.catino@oncologico.bari.it (A.C.); nicco.varesano@gmail.com (N.V.); galetta@oncologico.bari.it (D.G.); 3 Thoracic Oncology Unit, IRCCS, Istituto Tumori Giovanni Paolo II, 70124 Bari, Italy; 4 Section of Bari, National Institute for Nuclear Physics, 70126 Bari, Italy; angela.lombardi@ba.infn.it (A.L.); sonia.tangaro@ba.infn.it (S.T.); 5 Department of Physics, University of Bari Aldo Moro, 70126 Bari, Italy; roberto.bellotti@ba.infn.it; 6 Department of Soil, Plant and Food Science, University of Bari Aldo Moro, 70126 Bari, Italy

**Keywords:** breath analysis, Malignant Pleural Mesothelioma (MPM), Volatile Organic Compounds (VOCs), TD-GC/MS, machine learning, metabolic pathways

## Abstract

Malignant pleural mesothelioma (MPM) is a rare neoplasm, mainly caused by asbestos exposure, with a high mortality rate. The management of patients with MPM is controversial due to a long latency period between exposure and diagnosis and because of non-specific symptoms generally appearing at advanced stage of the disease. Breath analysis, aimed at the identification of diagnostic Volatile Organic Compounds (VOCs) pattern in exhaled breath, is believed to improve early detection of MPM. Therefore, in this study, breath samples from 14 MPM patients and 20 healthy controls (HC) were collected and analyzed by Thermal Desorption-Gas Chromatography-Mass Spectrometry (TD-GC/MS). Nonparametric test allowed to identify the most weighting variables to discriminate between MPM and HC breath samples and multivariate statistics were applied. Considering that MPM is an aggressive neoplasm leading to a late diagnosis and thus the recruitment of patients is very difficult, a promising data mining approach was developed and validated in order to discriminate between MPM patients and healthy controls, even if no large population data are available. Three different machine learning algorithms were applied to perform the classification task with a leave-one-out cross-validation approach, leading to remarkable results (Area Under Curve AUC = 93%). Ten VOCs, such as ketones, alkanes and methylate derivates, as well as hydrocarbons, were able to discriminate between MPM patients and healthy controls and for each compound which resulted diagnostic for MPM, the metabolic pathway was studied in order to identify the link between VOC and the neoplasm. Moreover, five breath samples from asymptomatic asbestos-exposed persons (AEx) were exploratively analyzed, processed and tested by the validated statistical method as blinded samples in order to evaluate the performance for the early recognition of patients affected by MPM among asbestos-exposed persons. Good agreement was found between the information obtained by gold-standard diagnostic methods such as computed tomography CT and model output.

## 1. Introduction

Malignant pleural mesothelioma (MPM) is a rare neoplasm mainly correlated to asbestos exposure. Asbestos mainly refers to six fibrous silicate minerals (chrysotile, amosite, crocidolite, anthophyllite, tremolite and actinolite) and it was widely used in building construction during the 20th century due to its strong chemical, abrasion and fire resistance [1,2]. In the last few decades, the production and the commercial use of asbestos have been banned in Europe [3,4,5] because it is known that asbestos is so carcinogenic that all asbestos fibers are classified in group 1 by the International Agency for Research on Cancer (IARC) of the World Health Organization (WHO) [6,7]. However, although the use of asbestos in developed countries has been banned since 2005, MPM is still a major public health issue. Indeed, its incidence is steadily increasing worldwide and substantial decreases are not expected before 2025 because of the long latency of the disease, up to 40 years between asbestos exposure and the onset of mesothelioma [8,9,10]. Moreover, the reported median survival for MPM is less than 1 year, with a 5-year survival rate below 5%. This dismal prognosis is mainly due to generally late diagnosis in advanced stages, and invasive diagnostic procedures such as a tissue biopsy obtained by thoracoscopy are often necessary to discriminate benign conditions from uncertain and/or neoplastic pleural lesions. Hence the need for a non-invasive and reliable method based on the identification of a disease-related biomarker or pattern of biomarkers correlated to the disease-risk [11].

In the last few decades, the use of a breath test based on the determination of VOCs concentrations has proven to be a promising and challenging research field for early detection of cancer. This screening method is non-invasive, simple, fast, safe for the patient and for the medical staff and could be a promising tool for screening and early diagnosis of MPM. In fact, it is well documented that asbestos inhalation determines the production of cytokine and reactive oxygen species (ROS), therefore inducing lipid peroxidation of the mesothelial cell wall, mutagenic DNA lesions and thus, malignant pleural mesothelioma [12]. VOCs deriving from these biological processes could be transported through the bloodstream to the lungs, where they enter the alveoli by the alveolar gas exchange mechanism and thus, are exhaled in the breath. Therefore, VOCs profile in breath may reflect changes in the subject’s health status [13,14,15].

However, to date very few studies have addressed determination of VOCs in human breath samples for MPM diagnosis. Among these, several studies were conducted using innovative, user-friendly and online technologies such as multicapillary column-ion mobility spectrometry (MCC/IMS) and sensors such as e-nose [16,17,18,19,20,21,22]. Dragonieri et al. [16] and Chapman et al. [17] were able to use e-nose to discriminate between MPM patients and controls with 92.3% and 90% sensitivity, analyzing 13 and 20 MPM patients’ breath samples, respectively. By using ion mobility spectrometry (IMS), Cakir et al. discriminated between 25 patients affected by asbestos-related diseases and 12 healthy controls (HC), with 99.9% accuracy [18]. In the same way, Lamote et al. analyzed breath samples collected from 23 MPM patients, 20 asbestos-exposed subjects and 21 healthy controls (HC) by using MCC/IMS, discriminating among different groups with 87% sensitivity [19]. Subsequently, the same research was extended to a larger data population discriminating between MPM and HC groups with 89% sensitivity [20]. Although easy to use, the instrumentations used in the above-mentioned studies were not able to identify VOCs or patterns of VOCs characterizing the disease. In fact, the gold standard analytical technique for this purpose remains gas-chromatography coupled with mass spectrometry combined with a thermal desorber (TD-GC/MS). To the best of our knowledge, only two studies have addressed the identification of VOCs in breath samples by using TD-GC/MS for early detection of MPM. Firstly, de Gennaro et al. discriminated between MPM patients and healthy volunteers with 97.4% accuracy starting from the analysis of breath samples collected from 13 MPM patients and 13 healthy not-exposed controls [21]. Secondly, Lamote et al. validated eNose against GC-MS, identifying VOCs in the exhaled breath of 14 healthy controls, 19 asymptomatic former asbestos-exposed (EXP) individuals, 15 patients with benign asbestos-related diseases (ARD) and 14 MPM patients [22]. Therefore, taking into account that MPM is a rare and aggressive neoplasm making patient recruitment difficult, this study aims to identify a distinct mesothelioma-related VOCs profile through breath analysis by developing and validating a promising data mining approach able to discriminate between MPM patients and healthy controls, even if no large population data are available.

## 2. Materials and Methods

### 2.1. Study Design and Participants

A cross-sectional and case-control study was conducted after approval by the Ethical Committee of the IRCCS-Istituto Tumori “Giovanni Paolo II” in Bari, Italy on 14 January 2019 (Prot. No. 679/CE). A total of 39 adult subjects aged between 49 and 82 were recruited from the Thoracic Oncology Unit after a computed tomography (CT) scan or chest radiograph confirming healthy conditions for all volunteers and before starting pharmacological treatment for advanced MPM patients. According to specific inclusion criteria, subjects affected by upper or lower respiratory tract infection during the last 4 weeks before the breath sampling, asthma, chronic obstructive pulmonary disease and systemic diseases, such as diabetes and malignancy, were not included in the study. As a consequence, the groups of recruited subjects were not completely matched for age due to difficulty in recruiting older volunteers without significant comorbidities as healthy controls. Demographic information and detailed data about medical conditions (potential comorbidities and pharmacological treatments), habits (smoking behavior) and previous asbestos exposure were collected and recorded on tailored questionnaires for all recruited volunteers (Table 1). All volunteers gave written informed consent before inclusion in the study and refrained from eating, drinking and smoking for at least 12 h before breath sampling.

### 2.2. Breath Sample Collection and Analysis

Exhaled breath collection was standardized for all subjects and was carried out in the same room of the Thoracic Oncology Unit where the volunteers remained for at least 10 min before breath collection so that an equilibrium was created between the lung and ambient air. More specifically, the volunteers breathed calmly through a mouthpiece with the nose clipped and slowly exhaled the full expiratory vital capacity. The procedure was repeated 2–3 times after 2 to 5 min of rest to fill the entire volume of the inert 3 L-Tedlar bags (Restek Corporation, Bellefonte, PA, USA). A statistical analysis was conducted before the study to evaluate the background VOCs of Tedlar bags used in this study and the most effective approach to reduce it. This consisted of filling ten different bags with wet high-purity grade air and measuring the VOCs content over the time and in different cleaning conditions. The VOCs background, mainly characterized by Phenol, CS_2_ and *N*,*N*-dimethylacetamide, drastically decreased after use. Moreover, memory effects were excluded when bags were flushed three times with high-purity grade inert gases and conditioned at a temperature higher than 40 °C. Therefore, in order to guarantee a minimum medium background, before using, bags were purged three times with high-purity grade air (S.I.A.D. S.p.A, Bergamo, Italy), then conditioned at 50 °C and finally flushed with another 3 L of ultrapure air. After breath collection, samples were either promptly transferred to a sorbent tube or sample bags were stored at room temperature and protected from direct sunlight and heat sources for a time period not longer than 1 h before the breath sample was transferred to a sorbent tube. In order to improve the breath analysis performance by making the analytical approach more specific for breath samples, the whole methodology, including VOCs sampling and pre-concentration on sorbent tube followed by thermal desorption and determination of VOCs by GC/MS analysis, was revised with respect to that reported in the previous works published by de Gennaro et al. [21,23]. In particular, more specific adsorbents for packing sorbent tubes and a cold trap were chosen in order to better manage wet samples. Moreover, a new chromatographic column and the optimization of TD-GC/MS parameters made it possible to analyze a wide range of VOCs useful for breath analysis. More specifically, two-bed sorbent tubes packed with Tenax TA and Carbograph 5 TD were used to collect exhaled VOCs (Bio-monitoring steel tube, Markes International Ltd., Llantrisant, UK), while a cold trap specific for wet samples (U-T4WMT-2S Water Management, Markes International Ltd., Llantrisant, UK) was used to trap organic compounds between ethane and C20 in a narrow band at the head of the column and a diphenyl dimethyl polysiloxane capillary column was used for VOCs speciation (VOCOL^®^-Supelco). Analysis of VOCs was carried out using a thermal desorber (TD) Unity 2 (Markes International Ltd., Llantrisant, UK) coupled with a gas chromatographer GC-Agilent 7890 and a mass spectrometer MS-Agilent 5975 (Agilent Technologies, INC. Santa Clara, CA, USA). The operating conditions of analysis are reported in Table 2. The GC-MS chromatograms were analyzed using the GCMS post-run analysis program and 103 VOCs were identified through spectral library matching (Compounds library of the National Institute of Standards and Technology, Gaithersburg, MD 20899-1070 USA) and through comparison with GC-MS chromatograms obtained by analysis of standard solutions of VOCs (Ultra Scientific Cus-5997). Among the identified VOCs, compounds linked to the Tedlar bags background were excluded, while VOCs related to ambient air contaminations, even if evaluated, were preliminarily used in data analysis in order to consider their potential contribution by discriminating between MPM patients and healthy controls, taking into account the hypothesis that exogenous VOCs could be differently catabolized by patients affected by cancer.

### 2.3. Statistical Data Analysis

Statistical data analysis was carried out using the R studio interface, version 3.6.1 (R foundation for statistical Computing, Vienna, Austria), starting from the peak area related to each detected VOC. A total of *p* = 103 compounds was identified in each chromatogram obtained from breath samples’ analysis. We applied a machine learning framework with three classifiers to discriminate the MPM patients from the controls. The aim of this approach is two-fold: (i) to investigate the predictive power of the compounds in classifying the two populations, and (ii) to identify the most predictive features among the total set of compounds.

**Figure 1:**
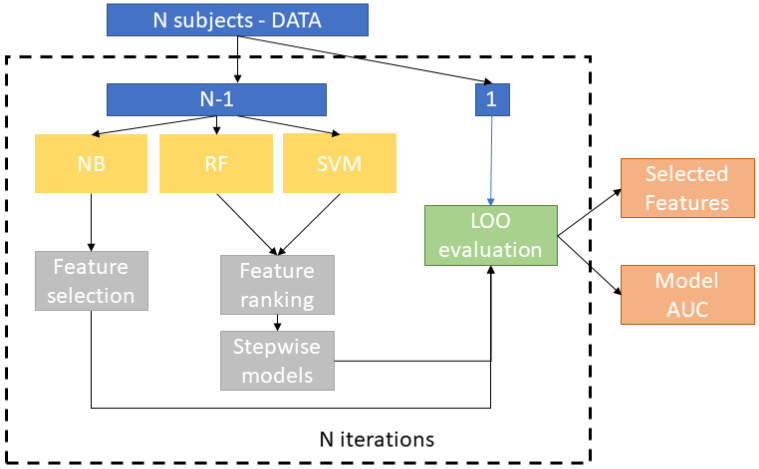
Overview of the machine learning framework.

An overview of the framework is shown in Figure 1. We applied a cross-validation scheme to quantify the predictive ability of the statistical model. In k-fold cross-validation, the whole dataset is partitioned into k parts with k analyses, where k − 1 folds are used for training while the omitted part is involved in the test. Here, a leave-one-out cross-validation (LOOCV) scheme was adopted. It represents a special case of k-fold cross-validation with k = N, N being the number of observations. This validation scheme is particularly appealing when the dataset is small in size in order to maximize the size of the training set. The application of LOOCV requires N analyses, each corresponding to a specific round of the cross-validation. In each round, we used N-1 samples to train three classifiers, select sub-samples of the compounds in order to identify the most significant set of compounds by means of a features’ selection technique and test the independent left out-of-sample subject. In particular, a “stepwise” training procedure was applied to evaluate the predictive power of the ranked subset of compounds. This approach involves a feature ranking algorithm and the evaluation of stepwise models trained for ranked subsets of increasing size (e.g., the top 5, 6, 7, and so on, up to P ranked features). It was introduced to identify the subset of features that maximizes the accuracy of the classification algorithm and it has proven to be an effective method in case of collinearity between features and high data dimensionality (i.e., P >> N) [24].

In this work, we compared three classifiers: Naive Bayes (NB) [25], Support Vector Machine (SVM) [26] and Random Forest (RF) [27]. These approaches are based on different assumptions and use distinct formulations to implement instance classifications. As an example, all the methods achieve high performance even with non-linear dependence between features, but SVM and RF are able to cope with the multicollinearity problem, unlike the NB method, which assumes independent variables.

In order to perform an exploratory analysis on the predictive features selected by the different classifiers, we chose algorithms with interpretable outcomes. Indeed, both filter and embedded feature selection techniques were implemented to select the most predictive features. Filters evaluate each feature without interaction with classifiers by using several criteria related to correlations among features or the amount of shared information, while embedded methods incorporate variable selection as part of the training process [28]. Both the SVM and RF models support embedded feature selection methods. We applied Recursive Feature Elimination (RFE) [29] for the SVM classifier and the Gini Index [30] for RF. For the Naive Bayes classifier, we applied a filter approach by using only the most significant features resulting from the Wilcoxon rank sum test in each iteration. It is worth noting that for all the methods, the output of the analysis is the number of non-redundant features to consider to yield the best performance of the models.

In order to evaluate the classification performance, the false positive rate (FPR) was computed as:

$$FPR=\frac{FP}{FP+TN}$$

and the true positive rate (TPR) as:

$$TPR=\frac{TP}{TP+FN}$$

where TP are the true positive samples, TN the true negative samples, FP are the false positives and FN are the false negative samples at the end of the N iterations. A receiver operating characteristic ROC analysis was carried out to highlight the accuracy of the classification algorithms at various threshold settings. We also computed the area under the ROC (AUC) as a comprehensive index of classification performance [31].

The machine learning framework provides a binary decision system with multivariate input and offers the important advantage of translating the binary decision value (i.e., 0/1) into a numerical probability score that identifies the probability that a given sample belongs to each of the two classes. In this work, we also used the ensemble of the best N models to evaluate the risk score for a group of exposed subjects. A consensus of the N probability scores were obtained to create the final score for each subject.

## 3. Results and Discussion

### 3.1. Discrimination between Malignant Pleural Mesothelioma Patients and Healthy Controls

**Figure 2:**
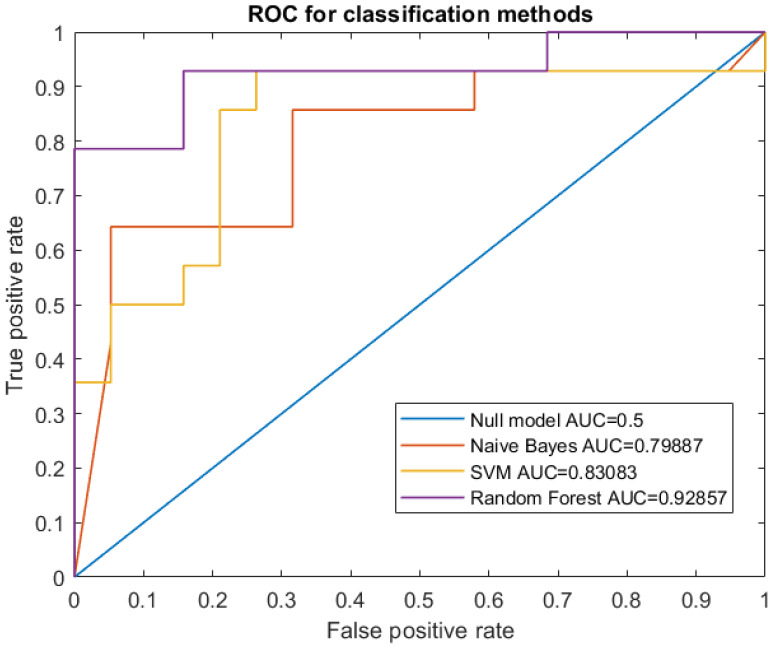
ROC curves of the best models for the three classifiers Naive Bayes, SVM and RF.

Figure 2 shows the ROC curves resulting from the best stepwise model of each classifier. The AUC values are reported in Table 3.

The Random Forest algorithm outperformed the other methods. This classifier is an ensemble learner of tree-structured base learners. Each tree individually predicts the target response while the final predictions result from the average of the individual tree predictions [27]. It is a nonparametric algorithm and the lack of a predefined form of the interactions between the features and the outcome variable allows the complex interactions between predictor variables to be modeled automatically.

These advantages have been exploited in many applications in different clinical scenarios, making this classifier one of the most popular for the development of computer-aided diagnosis (CAD) systems [32]. Since RF proved to be the best classifier among the three models, we investigated the most significant features ranked by the RF classifier. Indeed, an ensemble strategy should combine different classification approaches with comparable performance. In this case, the other two classifiers exhibit lower performance so they could prevail as a noise component in an ensemble logic resulting in a less reliable CAD system.

**Figure 3:**
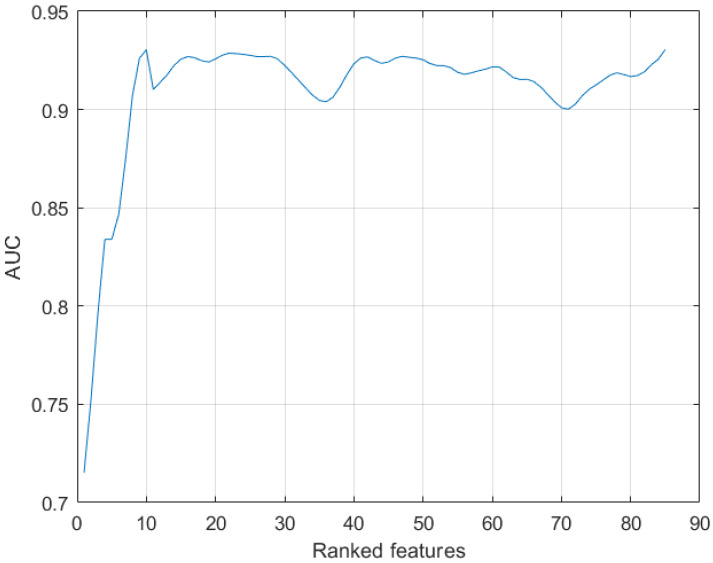
AUC values resulted from incremental ranked features of the RF model.

**Figure 4:**
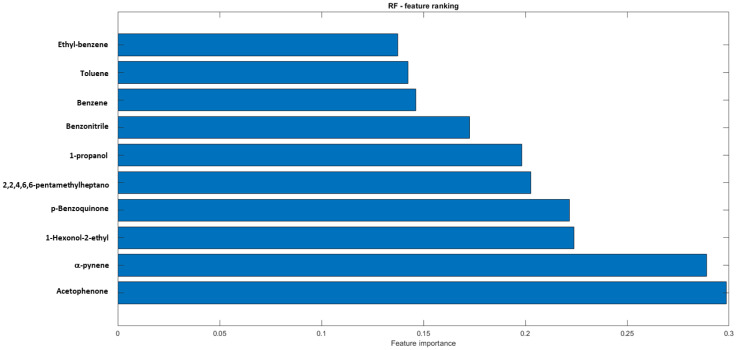
The first 10 ranked features of the RF classifier.

Figure 3 shows the AUC values resulting from incremental values of the number of features ranked by the RF model. As it can been noted, the first 10 features could be employed to reach the maximum value of performance. These features are listed in Figure 4.

In this study, a pattern of VOCs consisting of ketones, alkanes and methylate derivates, and hydrocarbons was found to be discriminate between MPM patients and healthy controls, with 93% accuracy. More specifically, as reported in Figure 4, the main VOCs identified as potentially diagnostics were acetophenone, α-pinene, 1-hexonol-2-ethyl, p-benzoquinone, 2,2,4,6,6-pentamethyl-heptane, 1-propanol, benzonitrile, benzene, ethylbezene and toluene. Although numerous studies have reported altered levels of VOCs in the breath of patients with lung cancer, to the best of our knowledge, only two studies have identified a VOCs pattern in breath specific enough to discriminate between MPM and healthy controls [18,21]. Moreover, even if the methodological approach used for breath sample analysis significantly affects the obtained results, some VOCs determined in our study as potentially diagnostic for MPM are coherent with those found by Lamote et al. [22] and de Gennaro et al. [21] in their studies focused on mesothelioma (acetophenone, α-pinene, 1-hexonol-2-ethyl, benzonitrile, toluene and 1-propanol). The other four VOCs, such as p-benzoquinone, 2,2,4,6,6-pentamethyl-heptane, benzene and ethylbezene, have been, to date, reported as potentially diagnostic in previous studies focused on lung cancer [14,18,21,33,34,35,36,37,38,39,40]. This result suggests that some VOCs found in human breath are common markers for both LC and MPM and this could be explained by considering that these VOCs probably originate from oxidative stress in the inflamed stroma. Moreover, among the studies on breath analysis, very few have been focused on the identification of metabolic pathways determining VOCs or altered patterns of VOCs in the breath of patients affected by cancer. In fact, to date, the association between cancer and the presence of specific VOCs in breath is reported as correlative or anecdotal, and the link between metabolic pathways altered by cancer and VOCs exhaled by patients affected by this pathology remain obscure [41]. This is even more the case for MPM that is a rare neoplasm still little researched. Therefore, for this study, we have reviewed several papers focused on biochemical processes probably activated by cancer in order to provide a comprehensive discussion on the potential metabolic pathways determining the presence of VOCs in breath samples among those found as markers of MPM in this study. It is easy to speculate that pathological processes such as metabolic disorders determined by cancer can produce new VOCs or change the ratio between the VOCs that are exhaled under normal conditions. It has been demonstrated that cancer alters or over-activates several metabolic pathways, such as glycolysis and oxidative stress, thus affecting the presence of VOCs in the different biological fluids. In fact, according to the well-known Warburg effect, even in aerobic conditions, cancer cells tend to favor metabolism via glycolysis rather than the much more efficient oxidative phosphorylation pathway [42,43]. This finding also explains the reason why the cancer cells survive and proliferate in a hypoxic microenvironment. De Berardinis and Fan conducted pioneering studies using ^13^C-glucose to investigate metabolic dysfunction of human lung cancer [44,45,46,47], demonstrating that, as compared to healthy tissue, in cancer cells, the glucose metabolism through glycolysis prevails over the full oxidation of glucose in the mitochondria due to increased production of ROS [48]. The high concentrations of mitochondrial ROS in the presence of lipids that can act as electron acceptors suggests that the mitochondria could be an important site of lipid peroxidation. In addition, mitochondria-derived ROS can diffuse into the cytosol and attack extra-mitochondrial lipids, as well as other molecules and, consequently, several VOCs could be released due to peroxidation of cancer-specific lipid species and/or due to ROS-mediated oxidation [49,50,51].

Regarding malignant pleural mesothelioma, it is well-know that asbestos is a fibrogenic and carcinogenic dust and that exposure to these mineral dusts induces the generation of reactive oxygen species that can damage macromolecules constituting cells such as phospholipids, proteins, enzymes and DNA, thus affecting physiological processes and/or causing cell death [48,49,50]. Therefore, the altered oxidative stress and the increased ROS concentration could produce high levels of oxidated organic species such as acetophenone, p-benzoquinone, propanol and 1-Hexonol-2-ethyl in human breath from MPM patients. For example, in several studies, acethophenone has been found to be a marker of lung cancer and MPM [14,20,21,33,52,53]. Moreover, Mamatha et al. found an increased level of acetophenone in the headspace of lung cancer cells as compared with cancer cell-free growth medium [40,54,55]. This finding could be linked to phenylalanine hydroxylase enzyme (PAH) deficiency due to oxidative stress, leading to the production of phenyl ketones by an alternative pathway to the main one, therefore determining the catalytic conversion of L-Phe to L-Tyr [46]. In addition, oxidative stress could also determine the conversion of benzene to phenol by means of oxygen and cytochrome P-450, and by further oxidation in the presence of cytochrome P-450 to hydroquinone and then, to p-benzoquinone [56,57,58].

Regarding alcohols, several studies have shown increased levels of 1-Hexonol-2-ethyl and 1-propanol in the headspace of different types of cancer cells as compared to the medium [36,38,39,40,52]. Moreover, 1-Hexonol-2-ethyl was also found exclusively in lung cancer patients’ saliva [59]. The presence of alcohols in breath is probably due to the metabolism of alkanes [40], that are partially excreted into the breath within minutes due to their low solubility in blood as well as to their oxidation to alcohols by cytochrome p450 (CYP450), a group of enzymes that are over-activated in cancer tissue [59]. As alkanes result from lipid peroxidation resulting from oxidative stress, the increased level of these two alcohols could be the result of increased oxidative stress and upregulated CYP450 [60,61].

Although hydrocarbon compounds such as benzene, ethylbenzene and toluene are exogenous pollutants related to exposure to tobacco smoke, pollution and radiation, several studies focused on breath analysis have found these VOCs as possible diagnostic biomarkers of cancer able to discriminate between cancer patients and healthy controls. It is reasonable to assume that patients affected by cancer have been exposed to excessive smoking and/or have experienced continuous occupational exposure to such exogenous compounds thus, up-taking these compounds into the fatty tissues of the body. These absorbed compounds could cause peroxidative damage to proteins, polyunsaturated fatty acids PUFA, and DNA, leading to age-dependent diseases such as cancer; furthermore, they might then be slowly and constantly released into the breath of patients affected by cancer. Even if hazardous compounds and xenobiotics in the body are first functionalized by the cytochrome p450 enzyme system, and then conjugated to a more soluble and excretable form by other enzyme systems, such as glutathione transferases, sulfotransferases and N-acetyltransferases, the altered catabolism of these compounds induced by cancer could probably determine their accumulation in the body and their release into the breath [37,62]. Even if benzonitrile and α-pinene have been identified among VOCs markers of MPM and lung cancer in several studies, further insights are needed to understand the metabolic pathway and/or the altered catabolic pathway induced by cancer for determining the presence of Benzonitrile and α-pinene in the breath of patients affected by lung and pleural neoplasms.

### 3.2. Independent Validation on Asymptomatic Former Asbestos-Exposed Subjects

Finally, in order to explore the potential application of the model proposed in this study for the early detection of patients affected by MPM among asbestos-exposed subjects, five breath samples collected from asbestos-exposed volunteers without symptoms were also exploratively analyzed and processed as blinded samples. We used the average value obtained from the test on models trained on MPM/HC classes to obtain a prediction score on asymptomatic former asbestos-exposed individual subjects. For each subject, the results were expressed in terms of the probability of belonging to the HC or the MPM class given by the RF classifier.

**Figure 5:**
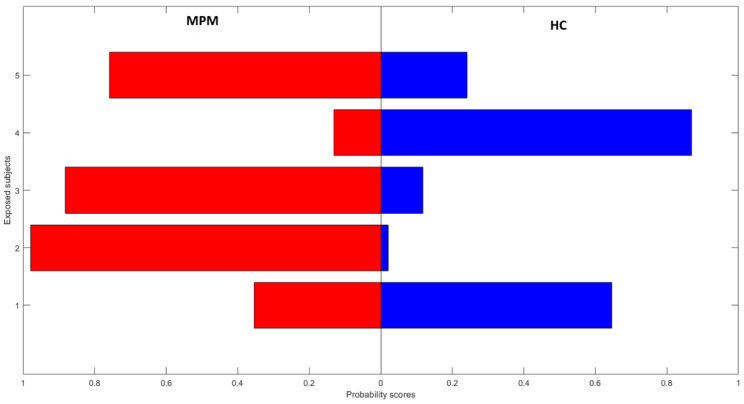
Probability scores for exposed subjects.

Figure 5 shows the probability scores for the exposed subjects belonging to the HC or the MPM class. Coherently with the statistical results, two of the five blind cases classified as “controls” by the model proved to be healthy after more accurate diagnostic examination by CT scan. The second blind case in Figure 5, classified as a pathological case with a probability score equal to 0.99, proved to be affected by MPM, as confirmed by CT scan. Furthermore, the patients corresponding to the third and fifth blind cases in Figure 5 were classified as pathological cases, probably due to the presence of pleural plaques visible on the CT scan. The presence of pleural plaques could represent a diagnostic challenge; in fact, this condition is generally correlated to asbestos exposure and its role as a risk factor for mesothelioma is still controversial. While taking into account the possible influence represented by the type of asbestos fibers and the exposure duration, as well as other factors such as smoking, numerous studies suggest that the presence of pleural plaques, together with their size and extent, may represent a risk factor for MPM [63,64,65,66]. Therefore, the group of asbestos-exposed subjects with pleural plaques could represent a different risk-group to be evaluated.

Although the study results are satisfying, we acknowledge some limitations. Firstly, as reported above, the MPM, AEx and HC groups were not matched for age. The patients affected by MPM were older than those in the other two groups due to the late diagnosis of MPM and because the recruitment of age-matched healthy controls without significant comorbidities is usually difficult. Even if age could be a confounding factor, some studies found that there was no correlation between aging and VOCs in breath [20,35,67,68]. However, considering the promising results obtained until now, the recruitment of patients and age-matched healthy controls is needed to optimize screening and improve the research output.

Secondly, we cannot fully exclude the possibility that external VOCs could have influenced the breath samples. However, in our opinion, the complete removal of environmental confounders is very hard to achieve [20,22,69], and the inclusion of exogenous compounds in data analysis could provide useful information about their kinetics, because inhaled VOCs, especially lipophilic compounds, can be stored in the body’s fat compartments and slowly released over time in different ways by patients and healthy controls [70,71]. Finally, we performed a preliminary analysis on five AEx subjects in order to explore the potential clinical application of the algorithm. We recognize that a new tailored model for a real-at-risk population should be developed and trained on more samples in order to capture the heterogeneity of asbestos-exposed persons, also considering the characteristics of asbestos exposure. However, the preliminary results obtained by exploring prediction scores on asymptomatic former asbestos-exposed individual subjects are encouraging enough to deserve the implementation of more reliable screening programs.

In summary, taking into account that early detection in MPM is very challenging, on the basis of the promising results obtained in this study, further research will be conducted in the framework of the first Apulian Breath Analysis Center to be set up in the South of Italy. This research will aim to extend this study on the external validation in a case-control series involving independent and in blind patient cohorts, as well as following at-risk subjects over time. This will allow the identification of the pattern of VOCs that is most responsive during the transition from chronic inflammation to malignant pleural mesothelioma, demonstrating the clinical utility of the breath test.

## 4. Conclusions and Future Perspectives

Taking into account that MPM is a rare and aggressive neoplasm and that the recruitment of MPM patients is very difficult due to late diagnosis, this study reported a promising data mining approach which was developed and validated in order to discriminate between MPM patients and healthy controls, even if large population data are not available. Three different statistical classifiers with a leave-one-out cross-validation approach were applied to test and validate the classification task, obtaining state-of-the-art results. Ten VOCs (ketones, alkanes and methylate derivates, and hydrocarbons) were able to discriminate between MPM patients and healthy controls. For each of them proving to be diagnostic for MPM, a potential metabolic pathway was analyzed and studied in order to link VOCs to the investigated neoplasm. Moreover, five breath samples from asymptomatic former asbestos-exposed (AEx) subjects were exploratively analyzed and processed as blinded samples in order to evaluate the performance of the model for the early recognition of patients affected by MPM among asbestos-exposed subjects. Interestingly, a good agreement was found between the information obtained by gold-standard diagnostic methods such as CT and the model output.

In conclusion, although the statistical approach in this study was developed and validated on a small data sample, our results agree with the little previous research conducted on MPM and with the majority of the results reported in the literature for lung cancer. The common markers detected in human breath samples collected by both LC and MPM patients indicate that these VOCs originate from oxidative stress in the inflamed stroma and thus, they are found in MPM, as in other cancers such as lung cancer. Moreover, the results obtained in this study suggest that breath analysis is a promising technique for the screening and early diagnosis of MPM due to its reliability and usefulness, as well as its non-invasive and easy-to-use characteristics. Thus, based on these promising findings, this study will be extended and further focused on the validation of our current results in an independent, large-scale, multicenter series, including the monitoring of AEx subjects over time, and also thanks to the recent institution of the first Apulian Breath Analysis Center in the South of Italy.

## Figures and Tables

**Table 1:** Patient characteristics.

Variation	MPM *	HC *	AEx *
Subject	14	20	5
Male/female	6/8	10/10	2/3
Age	73.6 (57–82)	53.6 (37–68)	63.5 (53–81)
Body Mass Index	24.9	24.0	24.4
BMI (Kg/m^2^)	(19.2–29.4)	(21.6–27.8)	(20.8–25.9)
Smoking status			
Current	0	3 (15%)	0
Ex	4 (29%)	4(20%)	2 (40%)
Never	10 (71%)	13 (65%)	3 (60%)
Pack/years	34.7 (19–62)	40.5 (21–73)	36.2 (32–55)

* MPM: malignant pleural mesothelioma patients; HC: healthy controls; AEx: asymptomatic former asbestos-exposed individual.

**Table 2:** Operative condition of TD-GC/MS analysis.

Step	Parameters	Value
Tube desorption	Purge time	3 min at 5 mL/min–trap in line
	Desorption time	10 min
	Desorption temperature	300 °C
	Temperature of cold trap	20 °C
	Desorption flow	30 mL/min, no split
Focusing trap desorption	Temperature of cold trap desorption	300 °C
	Split low	5 mL/min
	Transfer Line Temperature	200 °C
GC analysis	Gas carrier	He
	Gas flow	1.7 mL/min
	Analytical column	VOCOL^®^ (Supelco), diphenyl dimethyl polysiloxane with crosslinking moieties, 60 m × 0.25 mm ID, 1.5 μm stationary phase thickness
	Oven temperature	37 °C hold for 5 min
		37–190 °C at 6 °C/min
		190–200 °C at 2 °C/min
		220–220 °C at 15 °C/min
		220 °C hold for 3 min

**Table 3:** AUC values resulting from the three classification models.

Naive Bayes	SVM	RF
0.80	0.83	0.93
